# Effects of air abrasion with alumina or glass beads on surface characteristics of CAD/CAM composite materials and the bond strength of resin cements

**DOI:** 10.1590/1678-775720150261

**Published:** 2015

**Authors:** ARAO Nobuaki, YOSHIDA Keiichi, SAWASE Takashi

**Affiliations:** 1 - Nagasaki University, Graduate School of Biomedical Sciences, Department of Applied Prosthodontics, Nagasaki, Japan.; 2 - Nagasaki University Hospital, Clinic of Fixed Prosthodontics, Nagasaki, Japan.

**Keywords:** Composite resins, Cementation, Shear strength

## Abstract

**Objective:**

The study aimed to evaluate effects of air abrasion with alumina or glass beads on bond strengths of resin cements to CAD/CAM composite materials.

**Material and Methods:**

CAD/CAM composite block materials [Cerasmart (CS) and Block HC (BHC)] were pretreated as follows: (a) no treatment (None), (b) application of a ceramic primer (CP), (c) alumina-blasting at 0.2 MPa (AB), (d) AB followed by CP (AB+CP), and (e) glass-beads blasting at 0.4 MPa (GBB) followed by CP (GBB+CP). The composite specimens were bonded to resin composite disks using resin cements [G-CEM Cerasmart (GCCS) and ResiCem (RC)]. The bond strengths after 24 h (TC 0) and after thermal cycling (TC 10,000 at 4–60°C) were measured by shear tests. Three-way ANOVA and the Tukey compromise *post hoc* tests were used to analyze statistically significant differences between groups (α=0.05).

**Results:**

For both CAD/CAM composite materials, the None group exhibited a significant decrease in bond strength after TC 10,000 (p<0.05). AB showed significantly higher bond strength after TC 10,000 than the None group, while CP did not (p<0.05). GBB exhibited smaller surface defects than did AB; however, their surface roughnesses were not significantly different (p>0.05). The AB+CP group showed a significantly higher bond strength after TC 10,000 than did the AB group for RC (p<0.05), but not for GCCS. The GBB+CP group showed the highest bond strength for both thermal cyclings (p<0.05).

**Conclusions:**

Air abrasion with glass beads was more effective in increasing bond durability between the resin cements and CAD/CAM composite materials than was using an alumina powder and a CP.

## INTRODUCTION

In recent years, the demand for metal-free restorations has increased significantly owing to the changes in the aesthetic preferences of patients. Computer-aided design/computer-aided manufacturing (CAD/CAM) technologies have allowed the production of dental restorations through numerically controlled machining, resulting in uniform material quality, greater reproducibility, and a reduction in the production costs. These technologies have been used successfully with various ceramic materials. Further, composite blocks have recently been introduced as alternatives to conventional indirect or direct resin composites for crown restorations, as the former are cheaper and faster to produce^27^. It was found that CAD/CAM composite inlays exhibited significantly better color matching than did CAD/CAM ceramic inlays after 3 years of clinical service^7^. However, the long-term clinical performance, color stability, coefficient of thermal expansion, and surface roughness of CAD/CAM ceramics are better than those of CAD/CAM composite materials. On the other hand, CAD/CAM composite crowns exhibit better fracture resistance than do glass-ceramic crowns^10^ ^,^ ^16^. In addition, there are considerable advantages to using resins as restorative materials instead of glass-ceramic materials, since resins result in lower degrees of wear in the antagonist enamel^12^ ^,^ ^13^. Therefore, the recently introduced CAD/CAM composite blocks are considered suitable alternatives to glass-ceramic materials^8^ ^,^ ^21^. In general, using industrial polymerization processes involving high temperatures and pressures, one can fabricate CAD/CAM composite blocks^18^ with higher degrees of conversion and lower amounts of the residual monomer. As a result, the physical properties and color stability of these blocks are superior to those of conventionally polymerized resins^2^ ^,^ ^5^ ^,^ ^9^ ^,^ ^24^ ^,^ ^29^.

Resin cements are the materials of choice for the adhesive cementation of polymeric CAD/CAM composites^14^ ^,^ ^25^. For these materials, bonding can result from a chemical reaction, through mechanical retention, or from a combination of the two, and is strongly related to the composition of the resin composite cement used and the pretreatment to which the polymeric CAD/CAM composite is subjected. One of the most common methods of improving the mechanical retention of materials is to subject them to air abrasion with alumina^17^. This, in principle, cleans the surface of the polymeric CAD/CAM composite and simultaneously increases its surface area^23^. Depending on the composition of the polymeric material, it can be subjected to a chemical pretreatment involving silane coupling or the use of a bonding liquid^4^ ^,^ ^11^ ^,^ ^26^.

Alumina is the material used most commonly, in the form of airborne particles, for the primary abrasion of alloys^1^, zirconia^20^, and polymeric CAD/CAM materials^4^ ^,^ ^11^ ^,^ ^23^ ^,^ ^26^. Glass beads are used only for the air abrasion of enamel and dentin or nickel-chromium alloys^6^ ^,^ ^19^, since glass beads are softer than alumina. However, air abrasion with glass beads is not an effective pretreatment for improving the mechanical retention of materials. On the other hand, air abrasion with alumina can cause significant damage to the surfaces of CAD/CAM composite materials. There is limited information available on the effects of air abrasion with glass beads on the strength of the bonds between industrially polymerized CAD/CAM composites and resin cements.

Since CAD/CAM composite materials are also employed for long-term restorations, their adhesion characteristics have a marked effect on their durability. Therefore, in this study, we investigated effective methods for pretreating CAD/CAM composite materials by evaluating the suitability of air abrasion treatments performed using glass beads and comparing them with air abrasion treatments performed using alumina. Further, the effects of silane coupling on the durability of the bonds formed between resin cements and CAD/CAM composite materials were also investigated. The first hypothesis tested was that the use of glass beads for air abrasion does not damage the surfaces of CAD/CAM composite materials more than when alumina powder is used. The second hypothesis tested was that a pretreatment involving air abrasion with glass beads and subsequent silane coupling is effective in significantly improving the durability of bonds between resin cements and CAD/CAM composite materials.

## MATERIAL AND METHODS

**Figure 1:**
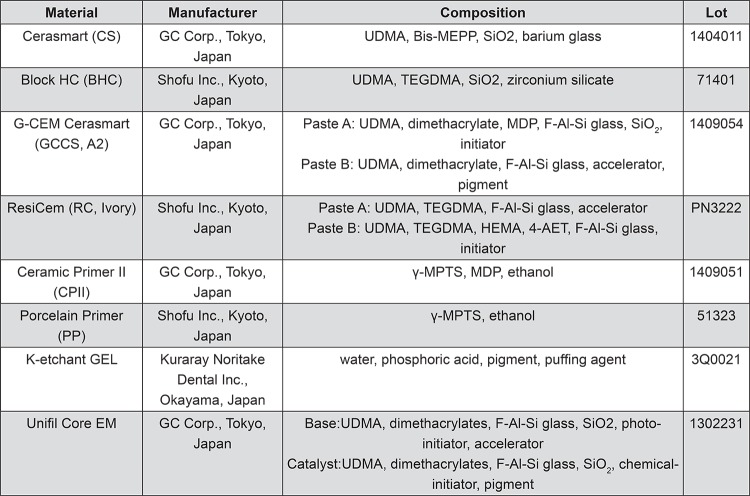
CAD/CAM composites, resin cements, and ceramic primers used in this study

UDMA: urethane dimethacrylate; Bis-MEPP: 2,2-bis(4-methacryloxypolyethoxyphenyl)propane; TEGDMA: triethylene glycol dimethacrylate; HEMA: hydroxyethylmethacrylate;

The following 2 CAD/CAM composite material systems were used in this study: 1) Cerasmart (CS), Ceramic Primer II (CPII), and G-CEM Cerasmart (GCCS); and 2) Block HC (BHC), Porcelain Primer (PP), and ResiCem (RC). Descriptions of the CAD/CAM blocks, resin cements, and ceramic primers investigated in this study are given in Figure 1. The CAD/CAM composite materials, resin cements, and ceramic primer were obtained from the same manufacturer.

4-AET: 4-acryloxyethyl trimellitic acid; γ-MPTS: γ-methacryloxypropyl trimethoxysilane;

MDP: 10-methacryloyloxydecyl dihydrogen phosphate

Blocks of the 2 CAD/CAM composite materials were cut into slices with dimensions of approximately 14×12×1.5 mm using a low-speed cutting saw with a diamond disk (Isomet, Buehler Ltd., Lake Bluff, IL, USA). The bonding surfaces of these plate-like specimens were ground using silicon carbide paper (#1,000, Struers, Ballerup, Denmark) under water cooling. They were then cleaned with phosphoric acid (K-etchant GEL) for 5 s, rinsed for 5 s, and dried with oil-free air for 5 s. Untreated specimens were used as controls.

### Air-particle abrasion protocols for determination of appropriate pressure

To determine the appropriate pressure for air abrasion using alumina (Hi-alumina, Shofu, Inc., Kyoto, Japan, mean particle size: 50 µm) and for air abrasion using glass beads (Glass Beads, Shofu, Inc., mean particle size: 75 µm), the following pressures were investigated: 0.1, 0.2, 0.3, 0.4, and 0.5 MPa. Air abrasion was performed using a blasting machine (Jet Blast III, J. Morita Mfg. Corp., Osaka, Japan), which was placed perpendicular to the surfaces of the plate specimens of the CAD/CAM composites at a distance of 10 mm for 15 s while the specified pressure was applied. The plates were abraded in circular movements to achieve a uniformly blasted surface. After being abraded, the plate specimens were cleaned with K-etchant GEL for 5 s, rinsed, and dried with oil-free air for 5 s.

Micromorphological examinations were performed on the specimens subjected to abrasion using the different particle types and air pressures by scanning electron microscopy (SEM) (SU-70, Hitachi High-Technologies Corp., Hitachinaka, Japan). Each specimen was sputter-coated with gold and analyzed at a magnification of 300×. The appropriate pressures for air abrasion for the 2 CAD/CAM composite materials were considered the ones for which the specimen surfaces did not exhibit damage at the filler, as determined from the SEM images.

### Surface roughness analyses

After the appropriate pressures for performing air abrasion using alumina and glass beads had been determined, the surface roughnesses of three specimens from each group were measured using a laser scanning microscope (VK-8500, KEYENCE Co., Ltd, Osaka, Japan) equipped with a 20× objective. The CAD/CAM composite blocks were placed and oriented appropriately on the stage of the microscope, and a laser beam with a spot size of 1 µm was used to scan their surfaces. The system used had submicron resolution along all the axes. Each surface was measured five times. The roughness values (Ra and Rz) were obtained using the Microsoft Windows-based Match software package.

### Preparation of bonding specimens

The specimens of the CAD/CAM composite materials were divided into 5 test groups (n=14), which were labeled as follows on the basis of the pretreatment method used:

Group None: Control group, which was cleaned with K-etchant GEL for 5 s, rinsed for 5 s, and dried with oil-free air for 5 s.

Group CP: A layer of a ceramic primer, obtained from the same manufacturer, was applied using a microbrush and dried in oil-free air for 5 s.

Group AB0.2: Air abrasion using alumina was performed at 0.2 MPa. This was followed by cleaning with K-etchant GEL for 5 s, rinsing, and drying in oil-free air for 5 s.

Group AB0.2+CP: The procedure for group AB0.2 was performed. This was followed by the procedure performed for group CP.

Group GBB0.4+CP: After the specimens had been subjected to air abrasion with glass beads at 0.4 MPa, their surfaces were cleaned with K-etchant GEL for 5 s, rinsed, and dried in oil-free air for 5 s. Subsequently, the procedure for group CP was performed.

A dual-polymerizing foundation composite resin (Unifil Core EM, GC Corp., Tokyo, Japan) was filled in acrylic plastic tubes (inner diameter of 6.0 mm and height of 2.0 mm) and polymerized in the curing apparatus (α Light II, J. Morita Mfg. Corp.) from both sides for 3 min each. The bonding surfaces of the resin composites were ground using silicon carbide paper (#1,000) under water cooling. They were then cleaned with K-etchant GEL for 5 s, rinsed for 5 s, and dried with oil-free air for 5 s.

Pieces of a polyethylene adhesive tape (approximately 50 µm in thickness) with a 4.0-mm-diameter circular hole were placed on the pretreated surfaces of the CAD/CAM composite plates to define the bonding area. The composite resin was bonded to every CAD/CAM composite plate with each of the resin cements by applying pressure using a finger. The excess cement was removed from the bonding margin using small disposable brushes. Light irradiation was performed by placing the tip of the light-emitting diode unit (power density of 1,000 mW/cm^2^; Pencure; J. Morita Mfg. Corp.) on the surface of the resin composite for 40 s. The bonded specimens were allowed to stand for 30 min at room temperature.

Each group was divided into 2 subgroups (n=7) corresponding to two different storage conditions. One subgroup was stored in distilled water at 37°C for 24 h. The other subgroup was subjected to 10,000 thermal cycles between water baths (Rika-Kogyo, Hachioji, Japan) kept at 4°C and 60°C; the dwelling time in each bath was 1 min.

### Shear testing procedure

Each bonded specimen was embedded in an acrylic resin mold and placed in an ISO/TR 11405 shear testing jig. The shear bond strength was measured with a universal testing machine (DCS-500, Shimazu Corp., Kyoto, Japan). The load was applied at a crosshead speed of 0.5 mm/min, with the bonding surface being parallel to the loading direction. The shear bond strength was calculated by dividing the force at which bond failure occurred by the bonding area.

The debonded surfaces were examined under an optical microscope (SMZ-10, Nikon Corp., Tokyo, Japan) to evaluate the failure types of the debonded specimens. The failures modes were classified into the following types: (i) adhesive failure (no resin cement remained on the CAD/CAM composite surface), (ii) mixed failure (some resin cement remained on the CAD/CAM composite surface and cracks formed within the CAD/CAM composite), and (iii) cohesive failure (failure occurred within the resin cement and cracks formed within the CAD/CAM composite or fracturing occurred within the CAD/CAM composite).

### Statistical analysis

SPSS 17.0 (SPSS Inc., Chicago, IL, USA) was used to analyze the results of the surface roughness and bond strength measurements. Two-way ANOVA tests and t-tests were performed on the surface roughness values in order to compare the differences corresponding to the air abrasion particles and CAD/CAM composite materials used. Three-way ANOVA tests and t-tests were performed on the bond strength values in order to compare the effects of the resin cements and the pretreatment methods used, as well as those of the 2 thermal cyclings. Tukey compromise *post hoc* tests were performed at a significance level of α=0.05.

## RESULTS

**Figure 2:**
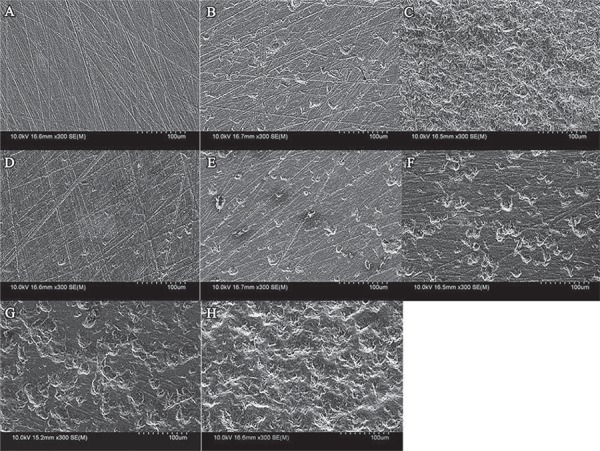
Scanning electron micrographs of the surfaces of the Cerasmart (CS) specimens after different pretreatments; A) #1,000 SiC paper (None); B) air abrasion with alumina powder at 0.1 MPa (AB0.1); C) air abrasion with alumina powder at 0.2 MPa (AB0.2); D) air abrasion with glass beads at 0.1 MPa (GBB0.1); E) air abrasion with glass beads at 0.2 MPa (GBB0.2); F) air abrasion with glass beads at 0.3 MPa (GBB0.3); G) air abrasion with glass beads at 0.4 MPa (GBB0.4); H) air abrasion with glass beads at 0.5 MPa (GBB0.5)

**Figure 3:**
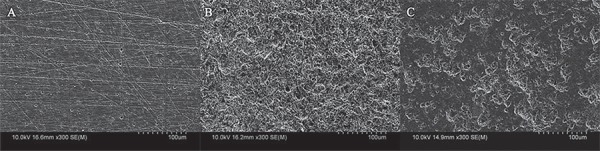
Scanning electron micrographs of the surfaces of the Block HC (BHC) specimens after different pretreatments; A) #1,000 SiC paper (None); B) air abrasion with alumina powder at 0.2 MPa (AB0.2); C) air abrasion with glass beads at 0.4 MPa (GBB0.4)

Images of the plate surfaces after they had been subjected to the different pretreatments are shown in Figures 2 and 3. The surfaces of the specimens of both CAD/CAM composite materials in group None (Figures 2A and 3A) exhibited small scratches; however, their structures were regular. Pretreatment AB0.1 resulted in a small number of defects (Figure 2B). Numerous concave and convex features formed on the surfaces of the CS specimens after pretreatment AB0.2 (Figure 2C). On the other hand, after the surfaces had been abraded with glass beads, the number of defects increased. Further, their depths increased with an increase in the air pressure from 0.1 to 0.4 MPa (Figures 2D-G). After the pretreatment of the GBB0.5 specimens (Figure 2H), their surfaces became irregular in a manner similar to those of the AB0.2 specimens; however, fewer irregular grooves were formed on their surfaces than on the surfaces of the specimens of the AB0.2 group. The surfaces of the BHC specimens pretreated with AB0.2 (Figure 3B) or GBB0.4 (Figure 3C) had morphologies similar to those of the surfaces of the CS specimens for AB0.1 and GBB0.1, 0.2, 0.3, and 0.5. However, the filler was observed falling out of the resin matrix in some of the specimens.

Table 1 lists the surface roughnesses of the specimens of the 2 CAD/CAM composite materials after the three pretreatments. The surface roughnesses of the specimens pretreated with AB0.2 or GBB0.4 were significantly higher than those of the None group (p<0.05). In contrast, no significant differences were found in the Ra values of the specimens of the CAD/CAM composite materials pretreated with AB0.2 and GBB0.4.

**Table 1:** Surface roughness of two CAD/CAM composite materials after different pretreatments

CAD/CAM Composite	Pretreatment	Ra (μm), Mean±SD	Rz (μm), Mean±SD
	None	0.97±0.10^a^	9.25±0.88^a^
Cerasmart (CS)	Air abrasion with alumina at 0.2 MPa (AB0.2)	2.32±0.13^b^	24.95±3.02^b^
	Air abrasion with glass beads at 0.4 MPa (GBB0.4)	2.36±0.16^b^	26.54±2.72^b^

	None	0.95±0.10^a^	10.32±1.67^a^
Block HC (BHC)	Air abrasion with alumina at 0.2 MPa (AB0.2)	4.09±0.37^c^	38.26±3.45^c^
	Air abrasion with glass beads at 0.4 MPa (GBB0.4)	4.30±0.30^c^	44.43±2.72^d^

Within the same column, the same superscript letters indicate no significant differences (p>0.05)

The mean shear strength values (and standard deviations) of the 2 resin cements to the CAD/CAM composite materials subjected to the various pretreatments are shown in Table 2. The None group specimens corresponding to the two resin cements exhibited decreased shear bond strengths after thermal cycling (p<0.05). The CP group specimens after TC 10,000 showed significantly higher shear bond strengths than did the None group for GCCS (p<0.05); on the other hand, the bond strengths for RC did not exhibit significant differences (p>0.05). The groups AB0.2, AB0.2+CP, and GBB0.4+CP corresponding to GCCS did not show significant differences in their shear bond strengths in the case of TC 0 and TC 10,000 (p>0.05). However, the GBB0.4+CP group showed the highest bond strength of all the groups for both thermal cyclings (p<0.05). The groups AB0.2+CP and GBB0.4+CP corresponding to RC did not show significant differences in their shear bond strengths after the 2 thermal cyclings (p>0.05). However, the AB0.2+CP group showed a significant decrease in shear bond strength after thermal cycling (p<0.05).

**Table 2:** Shear bond strength of two resin cements to CAD/CAM composite materials with different pretreatments

Resin Cement	Pretreatment for CAD/CAM Composite	Mean Shear Bond Strength±SD (MPa)	
		TC 0	TC 10,000
	None	25.6±2.9^cd,B^	13.9±1.6^b,A^
G-CEM	CP (CPII)	23.9±2.5^c,A^	23.4±2.1^c,A^
Cerasmart	AB0.2	28.4±3.2^de, A^	30.0±2.0^de,A^
(GCCS)	AB0.2+CP (CPII)	36.1±2.5^f,A^	32.7±4.4^ef,A^
	GBB0.4+CP (CPII)	41.0±3.6^g,A^	43.8±3.6^g,A^

	None	11.3±1.7^a,B^	8.1±2.3^a,A^
ResiCem	CP (PP)	19.6±2.0^b,B^	11.4±1.7^ab,A^
(RC)	AB0.2	29.8±3.0^e,A^	27.5±3.1^d,A^
	AB0.2+CP (PP)	40.3±1.4^g,B^	35.1±3.5^f,A^
	GBB0.4+CP (PP)	38.0±1.8^fg,A^	36.8±2.8^f,A^

The same small letters as superscript indicate no significant differences within the same column (same thermal cycling), and capital letters within the same row (same combination of resin cement and pretreatment) (p>0.05)

Table 3 shows the distribution of the failure types. All the specimens of the groups None and CP exhibited adhesive failures at the resin cement-CAD/CAM composite material interface for both resin cements. In contrast, no adhesive failures were observed in the groups AB, AB0.2+CP, and GBB0.4+CP, and only mixed and cohesive failures were detected. For the groups AB0.2+CP and GBB0.4+CP, cohesive failures were observed in most of the specimens after both thermal cyclings.

**Table 3:** Bonding fracture modes observed for different combinations of resin cement and pretreatment

Resin Cement	Pretreatment for CAD/CAM Composite	TC 0			TC 10,000		
		Adhesive	Mix	Cohesive	Adhesive	Mix	Cohesive
	None	7	0	0	7	0	0
G-CEM	CP (CPII)	7	0	0	7	0	0
Cerasmart	AB0.2	0	2	5	0	3	4
(GCCS)	AB0.2+CP (CPII)	0	1	6	0	2	5
	GBB0.4+CP (CPII)	0	0	7	0	0	7

	None	7	0	0	7	0	0
ResiCem	ACP (PP)	7	0	0	7	0	0
(RC)	AB0.2	0	1	6	0	3	4
	AB0.2+CP (PP)	0	0	7	0	2	5
	GBB0.4+CP (PP)	0	0	7	0	1	6

## DISCUSSION

The strength of the bond between the cement and the restoration, which determines the outcome of fixed dental restorations, depends on many factors^3^ ^,^ ^11^ ^,^ ^22^. A prerequisite for ensuring the longevity of industrially polymerized CAD/CAM composite materials, such as restorations, is to use a reliable pretreatment that allows for the long-term adhesion of the restoration to the cement. In this study, after the composite specimens had been subjected to pretreatment GBB0.4, they exhibited a lower degree of damage than that experienced by the specimens subjected to pretreatment AB0.2. With respect to the effects of the thermal cyclings, the specimens subjected to pretreatments GBB0.4 and CP exhibited significantly higher bond strengths than did those subjected to pretreatments AB0.2 and CP. On the basis of these results, the 2 tested hypotheses — that the use of glass beads for air abrasion results in a smaller degree of surface damage to CAD/CAM composite materials than that when alumina powder is used, and that pretreatments GBB0.4 and CP in combination are more effective in improving the bond durability of resin cements — were found to be true.

During the bonding process, the surface roughness of the material being bonded should be high enough to ensure adequate mechanical retention. In the present study, a number of pretreatments were used to improve the strength of the bonds between CAD/CAM composite materials and resin cements while ensuring that the surfaces of the materials experience the least amount of damage. Air abrasion with alumina powder is considered the most suitable pretreatment for cleaning and activating the restoration surface, as it thoroughly removes organic contaminants from the surface^28^. Moreover, alumina blasting increases the roughness of the bonding area to create micromechanical interlocks with the luting cement^17^. This mechanical retention is necessary for the bonding of industrially polymerized PMMA-based crowns^23^. Therefore, the adhesion between the alumina-blasted polymeric CAD/CAM composites and the resin cements could be considered mechanical retention. Polymeric blocks are industrially polymerized and exhibit a higher degree of conversion than do manually polymerized ones^14^. Since the control group with the untreated surfaces (group None) did not show durable bonding, it can be assumed that the free radicals were not sufficient for achieving adhesion between the resin cements and the surfaces of the polymeric CAD/CAM composites.

The microhardnesses of the CAD/CAM composite materials were much lower than those of the CAD/CAM ceramic blocks^14^. However, air abrasion with alumina powder has been used not only with metallic and ceramic materials^1^ ^,^ ^20^, but also with polymeric CAD/CAM materials^4^ ^,^ ^11^ ^,^ ^23^ ^,^ ^26^. The use of alumina for air abrasion may result in greater damage to the surfaces of CAD/CAM composite materials compared to that experienced by the surfaces of metals or ceramics. Therefore, we concluded that using glass beads instead of alumina powder is preferable for minimizing the damage to the surfaces of the materials. For both CAD/CAM composite materials, pretreatments AB0.2 and GBB0.4 increased the surface roughness to levels higher than that of the untreated group (None), with the composite materials exhibiting different surface morphologies, as determined using SEM. Pretreatment AB0.2 abraded the materials, creating a rugged surface over the entire specimen, while GBB0.4 resulted in smaller defects on the surface. Further, for both CAD/CAM composite materials, pretreatment AB0.2 resulted in greater damage to the surface than did GBB0.4, resulting in the falling out of the filler; this was particularly true for BHC. Damage to the cementation surface may lead to mechanical stress at the bonding area. As a result, cracks can form in the luting area. On the other hand, the surface roughnesses of both CAD/CAM composite materials when pretreated with GBB0.4 were not significantly different from those when they were pretreated with AB0.2. The high surface roughnesses of the CAD/CAM composite materials improved micromechanical retention and/or increased the degree of physical interaction with the resin cement.

Silane coupling is effective for improving bonding between silica filler-containing CAD/CAM composites and the resin monomer in resin cements. However, CP did not improve the bond strength of GCCS after TC 0. It also did not increase bond durability for BHC. Grinding the control group (None) specimens using silicon carbide paper did not expose the filler on the surface to a sufficient degree, as shown in Figures 2A and 3A. Therefore, the use of a silane coupling agent (CP) on the surface was ineffective in improving bond strength and bond durability. On the other hand, the bond durability for both resin cements was improved after pretreatment AB0.2. When pretreatments AB0.2 and CP were performed in combination, they were more effective than AB0.2 alone. Silane coupling agents promote the penetration of the luting agent into the surfaces of CAD/CAM composite materials to enhance micromechanical interlocking, and ultimately increase bond strength. When the surfaces of the CAD/CAM composite materials were pretreated with both GBB0.4 and CP, both the resin cements (GCCS and RC) showed higher shear bond strengths and bond durability than those when they were pretreated with AB0.2 and CP. Increasing wetting helps the luting cement effectively infiltrate the pores of the composite surface, thereby enhancing the bond strength^15^. After being subjected to pretreatment GBB0.4, the surface morphologies of the CAD/CAM composite materials exhibited a greater degree of wettability, making them more suitable for silane coupling and the penetration of resin cements than was the case after pretreatment AB0.2. This is probably what resulted in more of the specimens subjected to GBB0.4+CP exhibiting cohesive failures after TC 10,000 than those subjected to AB0.2+CP.

## CONCLUSIONS

Within the limits of the study, it may be concluded that air abrasion with glass beads at 0.4 MPa was more effective in increasing bond durability between the resin cements and CAD/CAM composite materials than using an alumina powder at 0.2 MPa and the subsequently application of a ceramic primer. Thus, air abrasion with glass beads can result in more durable bond strength between the resin cements and CAD/CAM composite materials, and causes a lower degree of surface damage than does air abrasion using an alumina powder.
